# Optimizing sampling across transect‐based methods improves the power of agroecological monitoring data

**DOI:** 10.1002/jeq2.20678

**Published:** 2025-03-17

**Authors:** Sarah E. McCord, Nicholas P. Webb, Justin W. Van Zee, Ericha M. Courtright, Ben Billings, Michael C. Duniway, Brandon L. Edwards, Emily Kachergis, Daniel Moriasi, Brian Morra, Aleta Nafus, Beth A. Newingham, Drew A. Scott, David Toledo

**Affiliations:** ^1^ USDA‐ARS Jornada Experimental Range Las Cruces New Mexico USA; ^2^ Bureau of Land Management, Colorado State Office, Denver Federal Center Lakewood Colorado USA; ^3^ Southwest Biological Science Center US Geological Survey Moab Utah USA; ^4^ Jornada Experimental Range New Mexico State University Las Cruces New Mexico USA; ^5^ Bureau of Land Management, Headquarters, Denver Federal Center Lakewood Colorado USA; ^6^ USDA‐ARS, MWA, National Laboratory for Agriculture and The Environment, Agroecosystems Management Research Unit Ames Iowa USA; ^7^ USDA‐ARS, Great Basin Rangelands Research Unit Reno Nevada USA; ^8^ Bureau of Land Management, National Operations Center, Denver Federal Center Denver Colorado USA; ^9^ USDA‐ARS Northern Great Plains Research Laboratory Mandan North Dakota USA

## Abstract

Transect‐based monitoring has long been a valuable tool in ecosystem monitoring to measure multiple ecosystem attributes. The line‐point intercept (LPI), vegetation height, and canopy gap intercept methods comprise a set of core methods, which provide indicators of ecosystem condition. However, users often struggle to design a sampling strategy that optimizes the ability to detect ecological change using transect‐based methods. We assessed the sensitivity of each of these core methods to transect length, number, and sampling interval in 1‐ha plots to determine: (1) minimum sampling required to describe ecosystem characteristics and detect change; and (2) optimal transect length and number to make recommendations for future analyses and monitoring efforts. We used data from 13 National Wind Erosion Research Network locations, including five LTAR sites, spanning the western United States, which included 151 plot sampling events over time across five biomes. We found that longer and increased replicates of transects were more important for reducing sampling error than increased sample intensity along fewer transects per plot. For all methods and indicators across biomes plots, three 100‐m transects reduced sampling error such that indicator estimates fell within a 95% confidence interval of ±5% for canopy gap intercept and LPI‐total foliar cover, ±5 cm for height, and ±2 species for LPI‐species counts. For the same criteria at 80% confidence intervals, two 100‐m transects are needed. Site‐scale inference was strongly affected by sample design, consequently our understanding of ecological dynamics may be influenced by sampling decisions.

## Core Ideas

- In rangeland, cropland, and pastureland ecosystems, plot‐level sample design impacts inference and uncertainty.
- Increasing sampling effort, particularly through longer length transects and increased numbers of transects, reduces plot‐level sampling error.
- At least two, but optimally three, 100‐m transects are recommended to reduce uncertainty across a 1‐ha plot.
- Plot design should be based on ecological processes of interest, ecological patterns, and uncertainty tolerance.

**Abbreviations**
AIM: Assessment, Inventory, and Monitoring
LoA: limits of agreement
LPI: line‐point intercept
NRI: National Resources Inventory
NWERN: National Wind Erosion Research Network

## 1 INTRODUCTION

Measuring ecosystem processes and functions is critical to understand ecological patterns and change (Ludwig et al., 2004). Transect‐based monitoring methods are a valuable tool in ecosystem monitoring globally to understand land health condition and trend, evaluate the effects of management, and plan future conservation efforts at both site and landscape scales (Densambuu et al., 2018; Kachergis et al., 2022; Spaeth et al., 2024; Webb et al., 2016). Transect‐based methods have been used to study woody species removal treatment effects (Bestelmeyer et al., 2019; Traynor et al., 2020), oil and gas reclamation (Lupardus et al., 2023), grazing regimes (Maestre et al., 2022), wind and water erosion (Webb et al., 2014), and ecological dynamics as influenced by management (e.g., Heller et al., 2022; Herrick et al., 2010; Miller et al., 2011). Transects can capture multiple ecosystem attributes to establish a multi‐faceted picture of ecosystem structure and function (Kachergis et al., 2022). For example, the line‐point intercept (LPI), vegetation height, and canopy gap intercept methods are a set of standardized, core methods, which together provide critical indicators of land condition across pasturelands, grasslands, shrublands, savannah ecosystems, and some croplands at over 85,000 locations globally (Herrick et al., 2018; McCord et al., 2023; Toevs et al., 2011; Webb et al., 2016). Common indicators calculated from these core methods include species richness and abundance, species cover, vegetation height and distribution, bare soil cover, and wildlife habitat characteristics (McCord, Brehm et al., 2022; Sofaer et al., 2022; Stiver et al., 2015). The core methods can also be used to build models predicting sediment transport by wind (e.g., Edwards et al., 2022) and water (e.g., Hernandez et al., 2017), as well as producing spatially explicit indicators of land cover using remote sensing (e.g., Allred et al., 2021; Gill et al., 2017; Rigge et al., 2020). Land managers, scientists, and other agroecosystem stakeholders use these indicators and models to understand ecosystem change across space and through time using benchmark values or ranges of values (Webb et al., 2024).

While the importance of these monitoring data is broadly acknowledged, producers, land managers, researchers, and others continually seek to reduce the costs of collecting monitoring data by sampling fewer plots across the landscape, decreasing the amount of data collected at a single plot, or both (Applestein & Germino, 2024; Herrick et al., 2017). Reduced data collection requirements can also have the benefit of expanding ecosystem monitoring and management partnerships, where stakeholders or citizen scientists with fewer monitoring may be more likely to participate in low‐intensity sampling efforts (Herrick et al., 2017; Riginos et al., 2011). However, reducing monitoring effort comes at a risk of increasing uncertainty in both plot and landscape inference. Therefore, it is critical to optimize monitoring programs to reduce error (or uncertainty) while minimizing sampling efforts in monitoring programs to ensure that ecosystem changes are reliably detected and not falsely identified due to error.

Error in monitoring programs can be partitioned into sampling error, or uncertainty due to sampling a subset of a population, and non‐sampling error, which describes all other sources of error (e.g., observer error; Elzinga & Salzer, 1998). Opportunities for reducing non‐sampling error in core methods studies are widely discussed and often transcend methods (Bonham, 2013; Elzinga & Salzer, 1998; McCord et al., 2021; McCord, Welty et al., 2022). Despite widespread adoption of the core methods, little information is available for practitioners to optimize sampling at the plot scale by reducing sampling error when using the LPI, canopy gap intercept, and vegetation height methods together. Although sampling error has been addressed for the LPI method (e.g., Bonham, 2013; Drezner & Drezner, 2021; Godinez‐Alvarez et al., 2009; Goodall, 1952; Herrick et al., 2005; Mueller‐Dombois & Ellenberg, 1974), these studies have been limited to a single ecosystem (e.g., Chihuahuan Desert; Herrick et al., 2005; Scarth, 2006) and focus solely on sampling intensity (i.e., number of pin drops along the transect; Drezner & Drezner, 2021; Scarth, 2006) or the number of transects per plot (Godinez‐Alvarez et al., 2009; Herrick et al., 2005). Other studies have explored sampling error in LPI due to pin drop placement, repeatability, and sample pin size (Bonham & Reich, 2009; Goodall, 1952). There is limited work exploring sampling error for the gap intercept method (Herrick et al., 2005) and vegetation height (Toledo et al., 2010). With insufficient information available on the optimal transect length and within‐transect sampling intensity, there is considerable variability among research projects and monitoring programs in the number, length, and sampling intensity of transects.

This variability in monitoring implementation is driven in part by the monitoring or study objectives, available sampling resources, and recommended best practices at the time the monitoring program was established (Bonham, 2013; Mueller‐Dombois & Ellenberg, 1974). For example, the US Natural Resources Conservation Service National Resources Inventory (NRCS NRI) program uses two intersecting 45.72‐m (150‐foot) transects with a total of 102 LPI pin drops, providing inference for 0.16‐ha plots (USDA NRCS, 2023); the US Bureau of Land Management Assessment, Inventory, and Monitoring (BLM AIM) program's standard implementation samples 0.28 ha using three 25‐m transects with a total of 150 LPI pin drops (Herrick et al., 2018); the Australian Terrestrial Ecosystem Research Network uses 10 100‐m transects with a total of 1000 LPI pin drops to sample 1 ha (White et al., 2012). Within the long‐term agroecosystem research (LTAR) network (Kleinman et al., 2018), sample designs range from one single 100 m with 100 LPI pin drops (Spiegal et al., 2024) to three intersecting 100‐m transects with a total of 1200 LPI pin drops to sample 1 ha at the US National Wind Erosion Research Network (NWERN) plots (Webb et al., 2016). Other studies and monitoring programs may use alternate transect and sampling configurations (Herrick et al., 2018).

In response to collective interest from land managers, ranchers, researchers, and other LTAR stakeholders seeking to both better characterize sampling error and potentially reduce monitoring costs, we assessed the sampling error of the LPI, vegetation height, and canopy gap methods using different transect lengths, number of transects per plot, and sampling intervals per transect. For each method, we determined (1) minimum sampling requirements to describe ecosystem characteristics and detect ecosystem change, and (2) the optimal transect length and number of replicates. We leveraged existing LTAR NWERN research plots to explore these questions as the network spans the western US agroecosystems, includes repeat measurements, and represents the upper limit of feasible data collection for most management and research projects. We then use these results to discuss considerations for analysis of existing monitoring data and to guide future monitoring efforts, drawing upon the collective expertise of the coauthors who represent both the research and land management communities.

**Core Ideas**

- In rangeland, cropland, and pastureland ecosystems, plot‐level sample design impacts inference and uncertainty.
- Increasing sampling effort, particularly through longer length transects and increased numbers of transects, reduces plot‐level sampling error.
- At least two, but optimally three, 100‐m transects are recommended to reduce uncertainty across a 1‐ha plot.
- Plot design should be based on ecological processes of interest, ecological patterns, and uncertainty tolerance.

## 2 MATERIALS AND METHODS

### 2.1 Study plots

**FIGURE 1:**
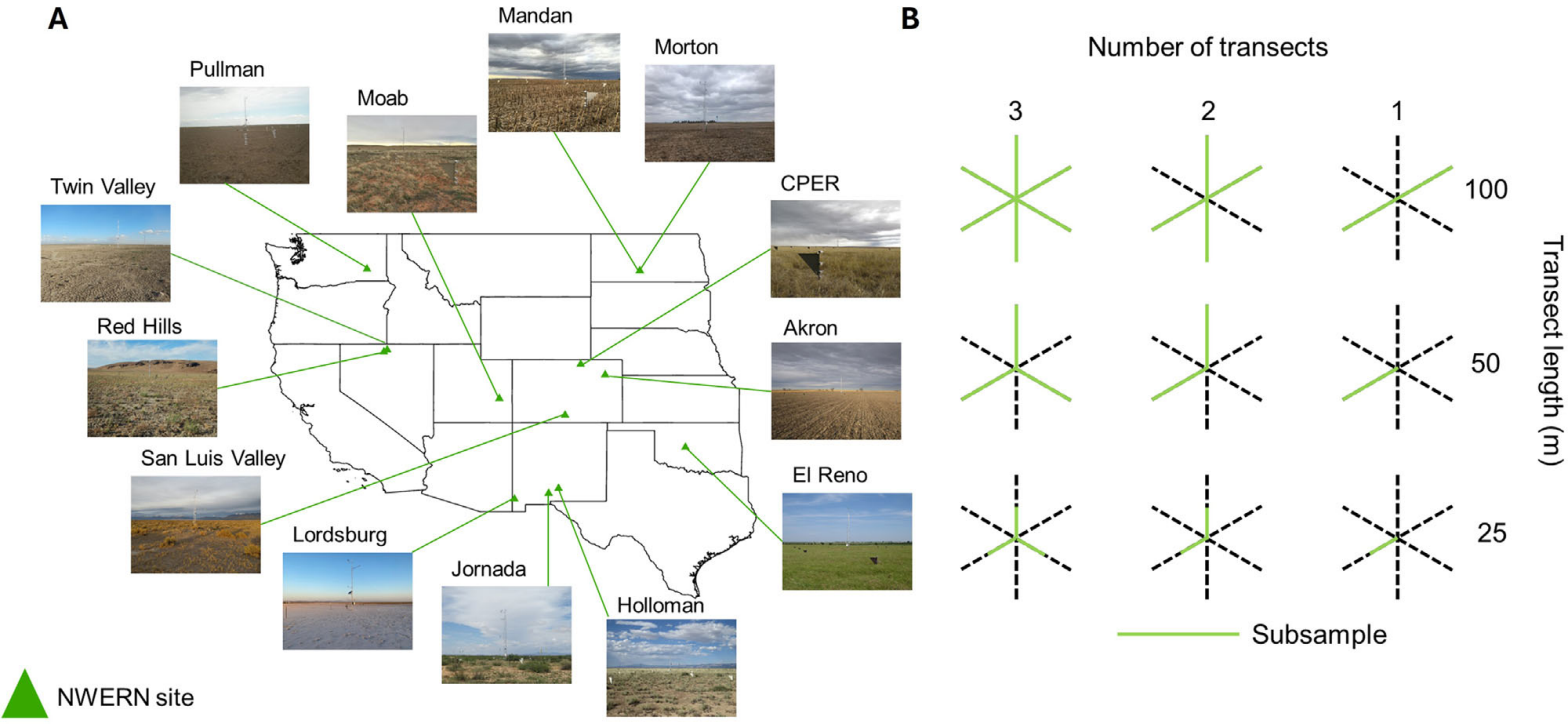
The 13 National Wind Erosion Research Network (NWERN) rangeland, pastureland, and cropland 1‐ha plots (A). At each site, three 100‐m radial intersecting transects were included in a full sample (B, top left). We then randomly subset the NWERN plots to represent nine scenarios of transect length and transect number. Subsampled areas are green, and the remaining unsampled area is in black dashes. For illustrative purposes, the transect are fixed directions; however, the subsampled transects were selected randomly.

We analyzed data collected by NWERN between 2015 and 2023 (Webb et al., 2016), including data from 13 plots, each with at least five data collection events, for a total of 151 sampling events (Figure 1). The NWERN plots represent a diversity of shrubland (Holloman, Jornada, Red Hills, San Luis Valley, and Twin Valley), grassland (CPER and Moab), pastureland (El Reno), cropland (Akron, Mandan, Morton, and Pullman), and playa (Lordsburg) ecosystems (Figure 1A, Table S1). Akron (cropland), CPER (rangeland), El Reno (pastureland), Jornada (rangeland), Mandan (cropland), Mandan (no‐till cropland), and Pullman (cropland) are located at LTAR sites network (Kleinman et al., 2018). Each NWERN plot represents 1 ha with a meteorological tower at the center (Webb et al., 2016). Three 100‐m transects intersect at the meteorological tower at 60° intervals (Figure 1B). Transect measurements follow Herrick et al. (2018): (1) canopy gap intercept between all plants (annuals and perennials) with a 5‐cm minimum gap, (2) LPI measured every 0.25 m, and (3) vegetation height measured every two meters. Data were collected one to four times per year, varying by location and data collector availability. All data collectors were trained and calibrated by NWERN staff. Data were accessed via the Landscape Data Commons (McCord et al., 2023).

### 2.2 Subsampling

All analyses were conducted in R 4.3.3 (R Core Team, 2024). To test the impacts of different sampling approaches, we subsampled the NWERN plots to create nine different sampling scenarios representing combinations of one to three transects of 25, 50, and 100 m (Figure 1B). First, by omitting data collected at certain locations along transect lines, we reduced transect length, where transect lengths <100 m were run from the plot center toward the edge of the plot (Figure 1B). Second, we reduced the number of transects from three to two or one by randomly omitting transects. Third, we reduced measurement intensity for LPI and vegetation height by omitting pin drops, resulting in pin drop measurement to produce intensities of 0.25‐, 0.5‐, 1‐, and 2‐m intervals for LPI and 2‐, 4‐, 6‐, 8‐, and 10‐m intervals for vegetation height. These subsampling scenarios were selected because they represent close approximations of common radial monitoring plot sample designs, such as BLM AIM (three 25‐m transects sampled every 0.5 m for LPI and every 2.5 m for height) and NRCS NRI (two 45.72‐m intersecting transects sampled every 91.44 cm for LPI and every 304.8 cm for height).

We calculated indicators for each subsampling scenario and method using the *terradactyl* R package (McCord, Brehm et al., 2022). For canopy gap intercept, we calculated the percent of the plot covered in all‐plant (live or dead) canopy gap size classes (5–24, 25–50, >50–100, >100–200, and >200 cm). We used the LPI observations to calculate percent total foliar cover and to count the number of species detected. Total foliar cover was selected as the primary indicator for LPI as it is a common vegetation‐based indicator available across rangeland and cropland agroecosystems and is studied in similar studies (e.g., Godinez‐Alvarez et al., 2009; Herrick et al., 2005). Furthermore, across NWERN sites, total foliar cover also represented a range of cover values, and therefore it captures concerns by detecting low and high plant cover attributes (0%–98.7%; Table S1). For vegetation height, we calculated the mean plant height of all rooted (live or dead).

### 2.3 Limits of agreement analysis

We conducted a limits of agreement (LoA) analysis to determine the effects of reducing sampling effort (compared to NWERN sampling) on indicator variables. LoA evaluates the differences between two measurement approaches with respect to their means (Martin Bland & Altman, 1986). In doing so, we determined the level of agreement between two approaches and detected bias exhibited by any one approach. For each indicator, we used the *SimplyAgree* R package (Caldwell, 2022) to conduct a nested LoA analysis that accounted for the repeat measures within NWERN plots, as well as the response across all plots using the MOVER method for calculating prediction intervals (Donner & Zou, 2012; Zou, 2013) at 80% and 95% confidence levels. We selected these confidence levels following Webb et al. (2019) and in response to our land manager coauthors who frequently use 80% confidence levels in analyses supporting decision‐making (e.g., M. G. Karl et al., 2016). All plots and indicators met the assumption of normality for the distribution of the differences. We compared the bias (systematic error) and agreement intervals (confidence interval) across sampling approaches and in comparison to acceptable difference criteria. These acceptable difference criteria match the acceptable difference limits for field crew calibration (Herrick et al., 2018), including (1) bias was not significantly different from zero, and (2) agreement intervals and their prediction intervals were ≤5% for total foliar cover and the canopy gap indicators, 5 cm for vegetation height, and two species for species count. We then identified the lowest effort sampling scenario required to meet the acceptable criteria at both 80% and 95% confidence levels for each of the core methods.

## 3 RESULTS

We evaluated nine sampling scenarios for transect length and number of transects for all core methods. Within these nine scenarios, we also evaluated different numbers of transects, four different measurement intervals for two LPI indicators (percent total foliar cover and species count), five canopy gap intercept size classes, and five different measurement intervals for vegetation height.

**FIGURE 2:**
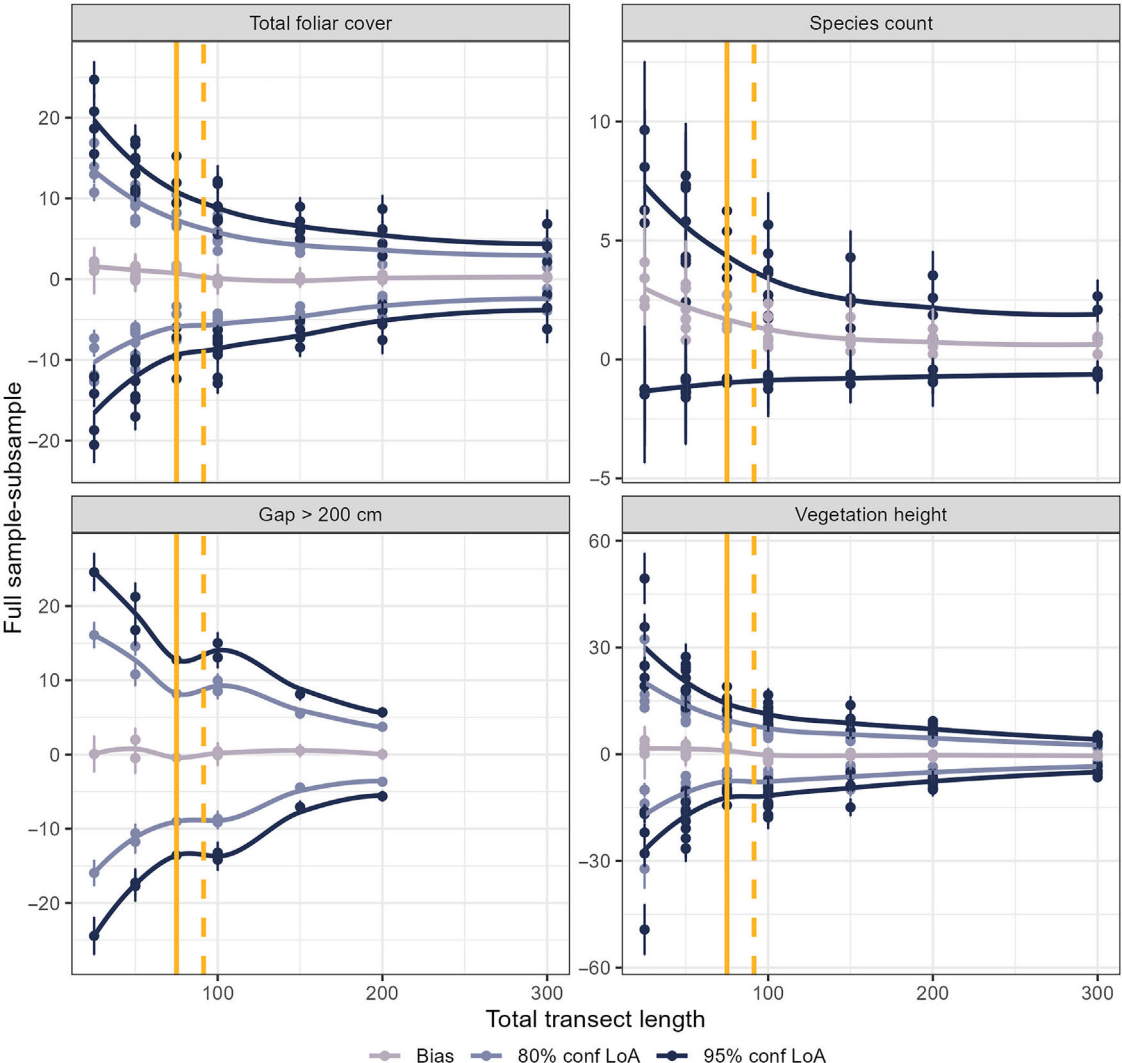
Limits of agreement intervals and bias for different total transect lengths at 95% confidence level. The solid yellow line represents the total transect lengths used in the Bureau of Land Management Assessment, Inventory and Monitoring program standard terrestrial design. The dashed yellow line represents the total transect lengths used in the Natural Resources Conservation Service National Resources Inventory program. LoA, limits of agreement.

**FIGURE 3:**
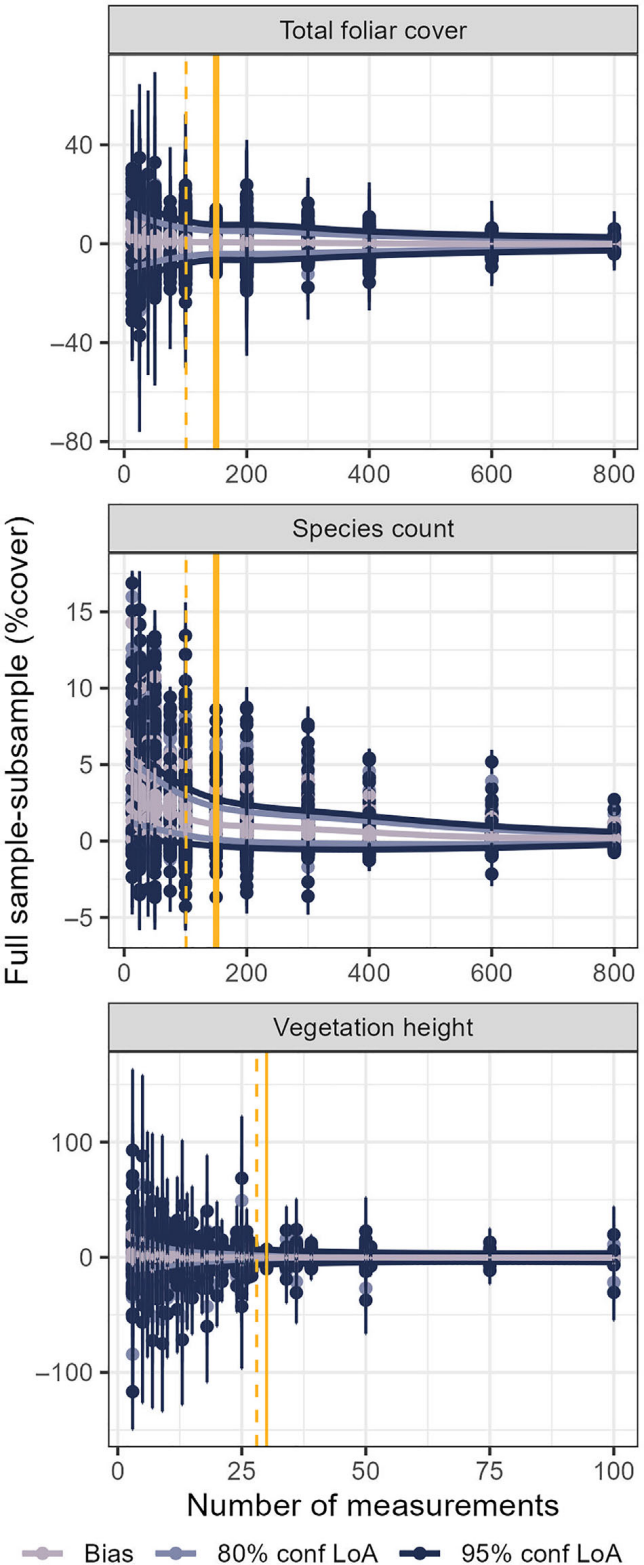
Limits of agreement intervals and bias for different numbers of measurements (i.e., pin drops) at 95% confidence level. The number of transects and transect length is variable. The solid yellow line represents the number of pin drops used in the Bureau of Land Management Assessment, Inventory, and Monitoring program. The dashed yellow line represents the number of pin drops used in the Natural Resources Conservation Service National Resources Inventory program. LoA, limits of agreement.

Sampling error for both LPI indicators decreased with longer transects and increased measurement intensities. The 95% confidence intervals for LPI‐total foliar cover ranged from 2.0% to 22.6%, while the 80% confidence interval ranged from 1.3% to 14.8% (Table 1, Table S1). Higher measurement intensity and 25‐m transects scenarios exhibited positive bias in both LPI indicators, showing that these scenarios underestimated the LPI indicators. Most subsampling scenarios were biased for LPI‐species count. For both LPI indicators, increasing combined total transect length (more and longer transects) had a greater effect on reducing the agreement interval (i.e., increasing precision) than increasing the sample intensity (Table 1, Figures 2 and  3). The minimum sample design to produce total foliar cover estimates within the 95% confidence interval is two 100‐m transects with measurements every 0.5, and to produce estimates within the 80% confidence interval is one 100‐m transect with measurements every 0.25 m, or three 50 m transects with measurements every 0.5 m to produce estimates (Table 2).

**TABLE 1:** The bias and agreement level from the limits of agreement (LoA) analysis for each sampling scenario within the LoA 95% confidence intervals.

		Number of transects	1			2			3		
Method	Interval	Transect length (m)	25	50	100	25	50	100	25	50	100
Gap intercept	5–24 cm	Bias	−0.28^*^	−0.2^*^	−0.08^*^	−0.38^*^	0^*^	−0.01^*^	−0.04^*^	−0.09^*^	–
		Agreement interval	4.32^*^	5.29	2.18^*^	3.91^*^	2.52^*^	1.2^*^	2.31^*^	2.78^*^	–
	25–50 cm	Bias	0.48^*^	0.08^*^	0.2^*^	−0.01^*^	−0.28^*^	0.14^*^	0.1^*^	−0.11^*^	–
		Agreement interval	7.14	6.11	4.54	5.03	4.39^*^	2.12^*^	4.48	2.16^*^	–
	51–100 cm	Bias	−0.15^*^	0.3^*^	0.12^*^	0.33^*^	−0.07^*^	−0.08^*^	0.12^*^	−0.04^*^	–
		Agreement interval	10.77	8.87	6.46	7.82	5.4	2.55^*^	6.04	3.56^*^	–
	101–200 cm	Bias	0.51^*^	−0.45^*^	0.75^*^	0^*^	1.07	0.17^*^	−0.03^*^	−0.23^*^	–
		Agreement interval	16.69	12.4	9.49	10.45	8.81	4.16^*^	8.43	5.22	–
	>200 cm	Bias	0.07^*^	2	0.42^*^	−0.48^*^	−0.07^*^	0.03^*^	−0.42^*^	0.53^*^	–
		Agreement interval	24.5	19.26	14.6	17.25	13.14	5.65	13.17	7.6	–
Vegetation height	2 m	Bias	1.53^*^	1.02^*^	−0.53^*^	1.4	0.42^*^	−0.36^*^	1.18^*^	0.3^*^	–
		Agreement interval	17.65	17.27	9.1	11.64	7.69	6.36	9.05	5.23	–
	4 m	Bias	1.46^*^	−0.53^*^	−1.52	1.76	0.43^*^	−0.09^*^	1.33	0.24^*^	−0.22^*^
		Agreement interval	23.48	18.29	9.28	12	9.29	5.24	11	6.35	2.93^*^
	8 m	Bias	2.41^*^	−0.55^*^	−2.14	2.22^*^	−0.54^*^	−0.56^*^	2.46^*^	−0.03^*^	−0.65^*^
		Agreement interval	19.11	25.87	12.46	15.71	17.24	7.98	13.35	8.74	4.22
	16 m	Bias	3.95^*^	0.36^*^	−0.48^*^	2.83	−0.51^*^	−0.08^*^	2.2	0.39^*^	−0.6^*^
		Agreement interval	31.85	24.01	13.61	18.62	13.65	9.39	12.51	9.6	5.85
	20 m	Bias	0.06^*^	0.42^*^	−0.72^*^	1.34^*^	−1.27^*^	−0.77^*^	2.34^*^	−0.55^*^	−0.16^*^
		Agreement interval	49.37	27	12.87	22.23	15.74	9.18	16.71	14.37	5.24
LPI—Total foliar cover	0.25 m	Bias	1.7	0.18^*^	−0.38^*^	1.46	−0.15^*^	−0.12^*^	1.08	−0.05^*^	–
		Agreement interval	13.8	10.5	5.92	11.59	7.35	2.98^*^	8.29	5.06	–
	0.5 m	Bias	2.23	0.54^*^	0.09^*^	1.63	−0.21^*^	0.26^*^	1.72	−0.16^*^	0.13^*^
		Agreement interval	16.41	10.57	7.2	11.51	7.69	4.15^*^	7.76	6.1	2.03^*^
	1 m	Bias	1.03^*^	0.12^*^	0.04^*^	1.2^*^	−0.23^*^	0.27^*^	1.16^*^	−0.06^*^	0.28^*^
		Agreement interval	19.74	14.6	8.98	13.82	9.15	5.91	10.78	7.22	3.81^*^
	2 m	Bias	2.09	−0.17^*^	−0.02^*^	1.16^*^	−0.56^*^	0.58^*^	1.44^*^	0.26^*^	0.34^*^
		Agreement interval	22.61	16.85	12.17	16.07	12.35	8.11	13.79	8.71	6.51
LPI—Species count	0.25 m	Bias	2.23	0.81	0.59	1.68	0.5	0.22^*^	1.26	0.34	–
		Agreement interval	3.51	1.6^*^	1.24^*^	2.5^*^	1.22^*^	0.65^*^	2.15^*^	0.97^*^	–
	0.5 m	Bias	2.51	1.31	0.78	1.72	0.9	0.53^*^	1.47	0.67	0.21^*^
		Agreement interval	3.76	2.74^*^	1.68^*^	2.6^*^	1.81^*^	1.33^*^	2.42^*^	1.7^*^	0.71^*^
	1 m	Bias	3.41	2.1	1.3	2.99	1.25	0.93	2.19	0.92	0.71
		Agreement interval	4.68	3.7	2.31^*^	4.34	2.49^*^	1.66^*^	3.2^*^	1.67^*^	1.37^*^
	2 m	Bias	4.08	2.98	1.79	3.18	2.35	1.29	2.73	1.78	0.96
		Agreement interval	5.56	4.22	2.66^*^	4.55	3.31^*^	2.25^*^	3.52	2.51^*^	1.7^*^

*Note*: Scenarios where bias is not significantly different from 0 and agreement intervals that are not significantly different from the agreement criteria (5% for canopy gap intercept and line‐point intercept [LPI] total foliar cover, 5 cm for vegetation height, two species for LPI species count) are noted in blue and starred. Dark blue indicates that both bias and agreement intervals were within the agreement criteria. Light blue indicates that only one of the two measures was within the acceptable limits. Dashes indicate maximum feasible sampling intensity, transect number, and transect length. See Table S2 for 80% confidence intervals.

* Significant within acceptable limits.

**TABLE 2:** Recommended plot sample design to detect change at the plot scale at a 95% confidence level and an 80% confidence interval, where the prediction interval is <5% for gap intercept and line‐point intercept total foliar cover, vegetation height is <5 cm agreement interval, and species count difference is <2.

		95% Confidence level			80% Confidence level		
Method	Indicator	Transect (*n*)	Transect length (m)	Interval (m)	Transect (*n*)	Transect length (m)	Interval (m)
Gap intercept	5–24 cm	1	25	–	1	25	–
	25–50 cm	2	50	–	1	50	–
	51–100 cm	3	50	–	2	50	–
		2	100	–	1	100	–
	101–200 cm	2	100	–	3	50	–
					2	100	–
	>200 cm	3	100	–	2	100	–
Vegetation height	Max height	3	100	4	3	50	4
					2	100	4
Line‐point intercept	Total foliar cover	2	100	0.5	1	100	0.25
					3	50	0.5
	Species count	2	100	0.5	2	100	0.5

The canopy gap intercept LoA intervals ranged from 1.2% to 24.5% at 95% confidence levels and 0.8% to 16.0% at 80% confidence levels (Table 1, Table S1). For all sampling scenarios, agreement intervals increased as gap size class increased. The subsampling scenarios were statistically unbiased for all but two sampling scenario‐indicator combinations: one 50‐m transect for gaps >200 cm and two 50‐m transects for gaps 101–200 cm. Overall, increasing the total transect length sampled decreased the size of the agreement interval. However, using two smaller transects, rather than one long transect, reduced both bias and agreement intervals—likely because site heterogeneity is better represented when measuring in multiple directions. The minimum sample design to ensure that all gap size class indicator estimates fell within the 95% confidence intervals was three 100‐m transects, whereas to produce gap estimates within the 80% confidence intervals, two 100‐m transects were required (Table 2).

Sampling error for vegetation height was also reduced by increasing transect length and measurement intensity (Table 1, Figures 2 and  3). The agreement interval range for the 95% confidence interval was 2.9–49.3 cm and 1.9–32.3 cm for the 80% confidence interval (Table 1, Table S2). Like the LPI indicators, increasing transect length was more effective for reducing agreement intervals and bias for vegetation height than was increasing the number of measurements, although this relationship was not as strong. The minimum sampling measurement interval for vegetation height at all confidence levels was 4.0 m, but three 100‐m transects were best for producing indicator estimates within the 95% confidence interval, versus two 100‐m transects or three 50‐m transects within the 80% confidence intervals (Table 2).

Similar to the LoA analysis across all plots, increasing total transect length and the sampling intensity decreased error and reduced bias at the individual plot level (Tables S3, Table S4, Figures S1–S5). However, there was no clear pattern according to ecosystem or management type. For all indicators, the Lordsburg plot had the smallest agreement intervals, with little variation in these intervals as sampling effort increased, which reflects the temporal and spatial homogeneity of the analyzed indicators at this playa plot with low cover and large canopy gaps. For LPI‐total foliar cover, five of 13 plots (Akron, CPER, Jornada, Lordsburg, Mandan, Moab, Morton, and San Luis Valley) had at least one subsampling scenario that met the acceptable criteria at 95% confidence levels (Table S4). For all‐plant canopy gaps >200 cm, only six of 13 plots (Akron, CPER, El Reno, Lordsburg, San Luis Valley, and Twin Valley) had at least one subsampling scenario that met the acceptable error criteria at 95% confidence levels (Table S4). For both height and species count, all plots had at least one subsampling scenario that met the acceptable error criteria at 95% confidence (Table S4).

## 4 DISCUSSION

Our study is the first to examine optimization among transect length, number of transects, and measurement intensity for LPI, all‐plant canopy gap intercept, and vegetation height across ecosystems. Increasing sampling effort reduced sampling error at all plots, with longer and increased numbers of transects being more important for reducing sampling error than increased sample intensity within transects. For all methods and indicators and across all plots, three 100‐m transects reduced sampling error such that indicator estimates fell within a 95% confidence interval to ±5% for canopy gap intercept and LPI‐total foliar cover, ±5 cm for height, and ±2 for LPI‐species counts. To produce indicator estimates that fall within an 80% confidence interval, two 100‐m transects are needed to adequately sample plot heterogeneity. These recommendations are less sampling effort than proposed in other studies (Herrick et al., 2005; Scarth, 2006; Yue et al., 2023), but still more effort than many regional and national monitoring programs (e.g., Densambuu et al., 2018; Kachergis et al., 2022; National Park Service, 2019; USDA Natural Resources Conservation Service, 2024). There are multiple considerations when selecting a plot‐level sampling approach, including data application, data collection costs, vegetation and soil patterns (and how they vary), and the consequences of missing a real change, or falsely identifying a change, in ecological dynamics due to sampling and non‐sampling error.

Our results show that sampling larger areas, especially plot layouts that represent at least 1 ha, is optimal. Due to data availability, it was not possible to evaluate the benefits (or lack thereof) for plots larger than 1 ha but the 1‐ha sampling area has been recommended in other rangeland sampling methods, including using remote sensing to describe ecological dynamics (Scarth, 2006; White et al., 2012), indicating larger areas are needed to capture the spatial heterogeneity typical of many ecosystems. However, shorter transects and smaller total plot areas may require less sampling effort and are less likely to be influenced by ecological variability driven by heterogeneity in soil and geomorphic properties that influence vegetation (Caudle et al., 2013). This is especially of concern in monitoring studies that rely on randomized sample designs to capture landscape‐level heterogeneity and may not have the leeway to move a plot to a homogeneous area.

The recommended transect length and total plot area sampled will also be driven by vegetation and soil patterns. For example, in ecosystems dominated by smaller plants (e.g., perennial grasses) that repeat at short intervals and have small canopy gaps, shorter transects may be appropriate as these features are equally well detected at short and long transect lengths because these are more fine‐grained features that have many opportunities to repeat. However, ecosystems such as shrublands with larger plants and larger (i.e., >2 m) canopy gaps that do not repeat as frequently may be difficult to represent using short transects, especially at lower sampling intervals for LPI and vegetation height (Drezner & Drezner, 2021; Pilliod & Arkle, 2013). In fact, for large canopy gaps, three 100‐m transects may not fully represent the variability of a 1‐ha plot, which is supported by Herrick et al. (2005), who suggested that a total of 450 m of transect is optimal. Other studies also found that spatial autocorrelation among LPI pin drops is particularly strong closer to the center of radial plot designs (Bonham, 2013; Mueller‐Dombois & Ellenberg, 1974; Yue et al., 2023) which may partially explain the increased bias we found on 25‐m transects. For this reason, some monitoring programs have a 5 m buffer between the plot center and the beginning of each transect (Herrick et al., 2018). If a monitoring study anticipates sampling across a range of vegetation patterns, or if it is possible that vegetation patterns might shift (i.e., from a grassland to a shrubland), then it may be necessary to implement larger plot areas to detect changes in heterogeneous ecosystems.

Increasing measurement intensity also decreased sampling error. Consistent with other studies (Drezner & Drezner, 2021; Godinez‐Alvarez et al., 2009; Herrick et al., 2005), across a range of cover amounts (Table S1), 400 pin drops are optimal for LPI sampling within the 95% confidence interval compared to 300–400 measurements within the 80% confidence interval. However, the incremental gains of increased precision begin to diminish around 200 LPI and 50 vegetation height measurements, which more closely reflects the sampling intensity selected for many land management monitoring programs (e.g., Densambuu et al., 2018; Kachergis et al., 2022; National Park Service, 2019). Although pin‐drop measures are theoretically statistically unbiased (Bonham, 2013), we found bias relative to the full NWERN sampling effort, particularly at shorter transect lengths. This indicates that where these pin drops are located with respect to both the plant clusters and individual plant features is important. Drezner and Drezner (2021) found pin drops should be spaced at intervals representing ∼80% of average plant width to best capture cover, although in clustered populations sampling error is reduced when the pin drop distance is greater than the size of the individual plants but smaller than the plant clusters (Lodge & Gleeson, 1979). Similarly, shorter transect lengths with more widely spaced measurement intervals were unbiased (albeit with larger uncertainties), in contrast to more frequent measurements. As expected, increasing the number of pin drops also increases the likelihood of detecting more plant species at all confidence levels. However, to detect rare and low‐cover species, we recommend supplementing transect‐based methods with a plot‐level species inventory (Drezner & Drezner, 2021; Herrick et al., 2018). This study did not explore measurement intensity (or minimum gap size) for all‐plant canopy gaps. However, where smaller gap sizes are common (and large gaps are likely to be rare in the future), sampling effort may be reduced by measuring shorter transects without increasing sampling error. Decisions about where to vary minimum gap size should be made in the context of other data (e.g., vegetation height and wind speed; Webb et al., 2021) as they relate to describing ecosystem processes (e.g., wind erosion; McCord et al., 2024).

As sampling error varies by plot sample design, it is important to keep the analysis objective in mind when designing a monitoring study or embarking on an analysis. Detecting small ecosystem changes may be difficult if uncertainty is greater than the desired ability to detect change. For example, if a treatment is considered effective with a 5% reduction in invasive plant cover, but the sampling uncertainty is ±7%, a manager is unlikely to infer treatment effectiveness correctly when the difference between treatment and control or treatment and a benchmark is <14%. Similarly, for analyses that rely on benchmarks (Webb et al., 2020, 2024), it is important to keep sampling uncertainty in mind when setting the benchmarks. For many rangeland ecosystems, wind erosion risk is higher when the cover of all‐plant canopy gaps larger than 1 m is >20% (Webb et al., 2014, 2024). If sampling uncertainty is 8% at 80% confidence levels, then a site may cross a functional threshold before the 20% threshold is detected by monitoring. This is particularly relevant for decisions that rely on a single plot to characterize an area and make a decision, such as oil and gas well pad reclamation release decisions (Lupardus et al., 2023). One solution is to include an “at‐risk” category that accounts for sampling uncertainty (e.g., 10%–20% >1 m gap cover) and identifies when additional lines of evidence are needed to evaluate if a threshold has been or is at risk of being crossed. If greater precision is needed to evaluate ecosystem changes, or if thresholds have been crossed, then monitoring studies will need to incorporate more intensive sampling to reduce uncertainty or explore more complex analytical approaches to account for error (e.g., Farnsworth & Bradley, 2013; Joseph & Bélisle, 2013).

Feasibility is an important consideration when designing monitoring programs and interpreting data. Feasible monitoring programs have greater durability and foster multi‐stakeholder collaborations to support monitoring and adaptive management (Derner et al., 2022; Herrick et al., 2017). It is important to explore the trade‐offs between monitoring objectives, available resources, and uncertainty. Identifying the acceptable confidence level is one such decision. Sample designs that ensure indicator estimates fall within the 95% confidence interval are more intensive and have lower sampling error than those where an 80% confidence interval is acceptable. One might also make sample design decisions with respect to the potential uncertainty decrease relative to the effort. For example, doubling sampling from two 50‐m transects to two 100‐m transects reduces the 95% confidence interval by more than half for all methods and sampling intensities. However, increasing sampling intensity for vegetation height and LPI on two 100‐m transects only reduces the confidence interval width by slightly more than 30% when increasing sampling intensity from 0.5 to 0.25 m for LPI‐total foliar cover. We also found that increasing sampling intensity does not fully compensate for the lost precision due to decreasing transect length for LPI; for example, precision is still lower for 300 LPI pin drops sampled at three 25‐m transects compared to 150 pin drops at three 50‐m or 3–100 m transects. Depending on objectives, reduced uncertainty (i.e., increased certainty) may not warrant the additional resources to increase sampling intensity. When weighing the feasibility of different monitoring plot designs, we also need to consider the interactions between sampling error and non‐sampling error. For example, when three 50‐m transects are sampled with LPI every 0.5 m versus two 100‐m transects every 0.25 m, there may be benefits to reducing observer error by laying out three shorter transects. Shorter transects may reduce observer fatigue by decreasing consecutive LPI measurements from 400 to 300, although it requires extra time to lay out additional transects. Understanding interactions between sampling error and non‐sampling error is critical to describing the overall quality of monitoring data (Biemer, 2010).

When designing monitoring studies, there are additional factors to consider. First, we explored sampling error for a small number of important indicators that occur across ecosystems susceptible to wind erosion, typical of many US croplands and rangelands. It is not known how well these results might extend to other indicators (e.g., bare ground, plant functional groups, plant production, biocrusts, and physical crusts), especially those that have small responses. Second, our plots do not represent all possible vegetation types and disturbance histories. Local analyses may be warranted if the ecotypes of interest differ significantly from the wind erosion susceptible ecosystems represented here (e.g., riparian areas that have dense vegetation). Third, this analysis considered single, intersecting, and spoke‐style transect layouts (Herrick et al., 2018). Different transect configurations (e.g., parallel transects) and directionality may impact uncertainty and bias in sampling across methods (Yue et al., 2023), although it has been hypothesized that unidirectional sampling may be vulnerable to bias toward, or away from, directional site characteristics (e.g., drill seedings or wind erosion “streets”; Gillette et al., 2006). We could not test the effects of alternate transect layouts in our study due to the predetermined radial plot design at NWERN plots. Finally, plot‐level sampling efforts may also be lessened to represent the broader landscape by dispersing transects further away from each other to represent a 1‐ha plot, but that is yet to be explored. Future research could explore transect layout and directionality effects on sampling error.

Here, we evaluated the impacts of sampling error at the plot scale. Herrick et al. (2005) found that decreasing plot‐level sampling from three 50‐m transects to one 50‐m transect doubled the number of plots needed across the landscape at the cost of increasing plot‐level uncertainty. It is unknown if this relationship holds with different transect lengths for all methods or sampling intensity for LPI and height. Additionally, for benchmark or categorical analysis, greater numbers of plots may not sufficiently overcome the costs of misclassifying the single plot as meeting or not meeting a desired condition (Joseph & Bélisle, 2013). The effects of plot‐level sampling uncertainty on landscape analysis may also vary by the landscape‐level sample design. It is likely more important to reduce uncertainty at targeted locations, which are selected for specific characteristics and are typically only represented by one plot, than at random locations that are intended to represent landscape‐level variability (J. W. Karl et al., 2017). Although this study examines the impact of sampling error from three monitoring study design variables (transect length, number of transects, and intensity of measurements) across a range of ecosystems, further exploration is needed to better understand the sampling error trade‐offs between allocating effort to more intensive plot‐level sample design versus sampling more plots across the landscape of interest.

## 5 CONCLUSION

Ecosystem monitoring is most effective when it is designed with stakeholder interest, management and research questions, and monitoring objectives in mind (Fischman & Ruhl, 2016). This includes acknowledging both the level of acceptable uncertainty in data‐supported decision‐making as well as the potential uncertainty present in monitoring data. We demonstrated across agroecosystems that plot‐scale inference is strongly affected by sample design, and consequently our understanding of ecological dynamics and the status, condition, and trend of ecosystems may be influenced by plot sampling decisions. Decisions regarding monitoring effort and plot design should be driven by (1) ecological process(es) of interest to land managers, stakeholders, and/or researchers including ecological responses to management; (2) amount of monitoring resources available; (3) ecosystem spatial patterns and expected changes over time; (4) tolerance for sampling error; and (5) a consideration for monitoring data needs in response to future management priorities or ecological shifts. This study also highlights the benefits of the LTAR network adopting standardized monitoring already used by the network's stakeholders. We show that intensive sampling is suggested at LTAR and NWERN sites to reach a greater confidence level (e.g., 95%) and lower sampling errors, as researchers seek to describe ecosystem processes such as wind erosion to develop decision support tools (Edwards et al., 2022; Webb et al., 2017). LTAR stakeholders benefit from the methodological and ecological insights of this additional sampling. When LTAR decision support tools are implemented by stakeholders, for whom monitoring resources are more limited and greater uncertainty may be acceptable, less intensive sampling may be appropriate. Because the data are collected using standardized methods, methodological and ecological insights from LTAR can be directly transferred to stakeholders to support adaptive management and monitoring (e.g., Webb et al., 2024). In this study, we leveraged LTAR and NWERN monitoring investments to provide improved information to those designing monitoring studies and analyzing monitoring data to identify the appropriateness of sample designs to meet monitoring objectives. This study demonstrates that, where possible, more intensive sampling is warranted to increase precision and reduce bias. Appropriate sample design will ensure that we are neither over‐ nor under‐allocating the extensive resources needed to monitor ecosystem change.

## AUTHOR CONTRIBUTIONS

**Sarah E. McCord**: Conceptualization; data curation; formal analysis; investigation; methodology; software; visualization; writing—original draft; writing—review and editing. **Nicholas P. Webb**: Conceptualization; writing—original draft; writing—review and editing. **Justin W. Van Zee**: Data curation; methodology; writing—original draft; writing—review and editing. **Ericha M. Courtright**: Data curation; writing—original draft; writing—review and editing. **Ben Billings**: Data curation; writing—review and editing. **Michael C. Duniway**: Conceptualization; writing—original draft; writing—review and editing. **Brandon L. Edwards**: Writing—original draft; writing—review and editing. **Emily Kachergis**: Writing—original draft; writing—review and editing. **Daniel Moriasi**: Writing—original draft; writing—review and editing. **Brian Morra**: Writing—original draft; writing—review and editing. **Aleta Nafus**: Writing—original draft; writing—review and editing. **Beth A. Newingham**: Data curation; writing—original draft; writing—review and editing. **Drew A. Scott**: Data curation; writing—original draft; writing—review and editing. **David Toledo**: Data curation; writing—original draft; writing—review and editing.

## CONFLICT OF INTEREST STATEMENT

The authors declare no conflicts of interest.

## Supporting information




**Table S1** The National Wind Erosion Research Network (NWERN) plots used by agroecosystem type, the number of sample events (*n*), and the first and last date of data collection.
**Figure S1**. Species detection curves from the line‐point intercept method for each National Wind Erosion Research Network (NWERN) site for increasing numbers of measurements (A) and total transect length (B).
**Figure S2**. Limits of agreement intervals and bias for total foliar cover for different total transect lengths and numbers of measurements at 95% confidence level.
**Figure S3**. Limits of agreement intervals and bias for species counts derived from the line‐point intercept method at different total transect lengths and numbers of measurements at 95% confidence level.
**Figure S4**. Limits of agreement intervals and bias for mean vegetation height derived from vegetation height method at different total transect lengths and numbers of measurements at 95% confidence level.
**Figure S5**. Limits of agreement intervals and bias for different total transect lengths at 95% confidence level for all‐plant canopy gaps >200 cm.

**Table S2** The bias and agreement level from the limits of agreement (LoA) analysis for each sampling scenario at each site within the LoA 80% confidence intervals.

**Table S3** The bias and agreement level from the limits of agreement (LoA) analysis for each sampling scenario at each site within the LoA 80% confidence intervals.

**Table S4** The bias and agreement level from the limits of agreement (LoA) analysis for each sampling scenario at each site within the LoA 95% confidence intervals.

## Data Availability

Data and code used in this study are available on the AgDataCommons (https://doi.org/10.6073/pasta/b8b8abc0471c826c755788c3f24a2c1b) and on the Landscape Data Commons (https://www.landscapedatacommons.org).
