# Supplementary material for: Optimizing sampling across transect‐based methods improves the power of agroecological monitoring data
Source: J Environ Qual. 2025 Mar 17;54(3):706–19. doi: 10.1002/jeq2.20678 (PMC12065049; doi:10.1002/jeq2.20678)
Supplement: Supplementary file 1 — Table S1 The National Wind Erosion Research Network (NWERN) plots used by agroecosystem type, the number of sample events (n), and the first and last date of data collection. Figure S1. Species detection curves from the line‐point intercept method for each National Wind Erosion Research Network (NWERN) site for increasing numbers of measurements (A) and total transect length (B). Figure S2. Limits of agreement intervals and bias for total foliar cover for different total transect lengths and numbers of measurements at 95% confidence level. Figure S3. Limits of agreement intervals and bias for species counts derived from the line‐point intercept method at different total transect lengths and numbers of measurements at 95% confidence level. Figure S4. Limits of agreement intervals and bias for mean vegetation height derived from vegetation height method at different total transect lengths and numbers of measurements at 95% confidence level. Figure S5. Limits of agreement intervals and bias for different total transect lengths at 95% confidence level for all‐plant canopy gaps >200 cm. [file JEQ2-54-706-s002.docx]

**Supplement 1: National Wind Erosion Research Network plot level results**

Table S1.1 The National Wind Erosion Research Network (NWERN) plots used by agroecosystem type, the number of sample events (n), and the first and last date of data collection. Median (min-max) values for Total Foliar cover, Height, and four gap indicators are also presented. For further description of each site, see https://winderosionnetwork.org .

| Plot | Type | n | First Visit | Last Visit | Total Foliar Cover (%) | Height (cm) | Gap 25 - 50 cm (%) | Gap 51 -100 cm (%) | Gap 101-200 cm (%) | Gap > 200 cm (%) |
| --- | --- | --- | --- | --- | --- | --- | --- | --- | --- | --- |
| Akron* | Cropland | 8 | 12/21/2018 | 10/4/2021 | 77.5(12.3-96.1) | 13.9(4.6-62.5) | 1.3(0-2.7) | 0.5(0-2.1) | 0.3(0-2.8) | 0.7(0-1.7) |
| CPER* | Grassland | 11 | 4/14/2016 | 8/21/2019 | 87.1(78.8-95) | 37.4(24.6-54) | 0(0-0.8) | 0(0-0.2) | 0(0-0.5) | 0(0-0.9) |
| El Reno* | Grassland | 5 | 6/19/2017 | 12/10/2019 | 86.8(79.3-98.7) | 40.7(17.6-54.5) | 0.1(0-2) | 0(0-1.1) | 0(0-1.3) | 0(0-4.7) |
| Holloman | Shrubland | 24 | 5/18/2015 | 3/28/2023 | 30.4(23.7-56.8) | 34.9(22.7-55) | 7.3(5.6-8.6) | 15.6(12.5-19.4) | 24.4(21.4-28.8) | 27.8(21.5-32.8) |
| Jornada* | Shrubland | 25 | 5/5/2015 | 3/23/2023 | 21.7(13.6-33.3) | 31.9(23.7-39.6) | 4(1.4-12) | 10.7(4.6-21) | 20.3(10.1-27.3) | 43(14-70.6) |
| Lordsburg | Playa | 6 | 9/2/2020 | 6/8/2022 | 0(0-0.1) | 1.4(1-2.3) | 0(0-0) | 0(0-0) | 0(0-0) | 98.8(98.8-98.8) |
| Mandan | No-till cropland | 11 | 8/3/2015 | 10/31/2019 | 22.3(0.6-88.3) | 23.6(6.9-144.2) | 3.3(0-20) | 7.3(0.8-27.3) | 3.8(1-23.4) | 13.1(0-97.7) |
| Moab | Grassland | 14 | 5/5/2016 | 2/23/2021 | 26.9(16.9-41.2) | 24.3(18.7-32.4) | 9.4(1.6-22.4) | 17.6(5.1-29.8) | 23.1(7.8-33.1) | 25.8(1.5-70.8) |
| Morton | Cropland | 7 | 5/31/2019 | 7/19/2021 | 59.5(6.9-86) | 23.7(10.1-128.1) | 2(0-19) | 2(0.2-17.6) | 1.6(0-27.8) | 2.8(0-76.3) |
| Pullman* | Cropland | 10 | 10/28/2016 | 10/5/2020 | 26.8(0-72.5) | 18.1(11.6-42.5) | 6.9(0-18.9) | 6.5(0-11.6) | 7.7(1.4-16.6) | 17.8(6.5-96.4) |
| Red Hills | Post-fire shrubland | 8 | 7/17/2019 | 8/25/2022 | 27(18.4-36.4) | 19.9(13.8-30.3) | 15.8(14.2-21.3) | 24.3(14.5-28.6) | 23(7-29.4) | 6.2(3.3-13.1) |
| San Luis Valley | Shrubland | 13 | 6/7/2016 | 10/2/2020 | 34.9(26.1-51) | 20.5(17.1-23.1) | 14.1(7.8-22.2) | 20.9(16.2-25.5) | 19.8(8.9-34.5) | 9.3(0.8-25.1) |
| Twin Valley | Post-fire shrubland | 9 | 6/12/2019 | 10/20/2022 | 25.9(5.3-62.7) | 26.4(6.8-33.2) | 7.4(1.8-20.2) | 10.3(5.3-18.7) | 16.5(0-22.5) | 43.5(0-73.8) |
| *LTAR sites |  |  |  |  |  |  |  |  |  |  |


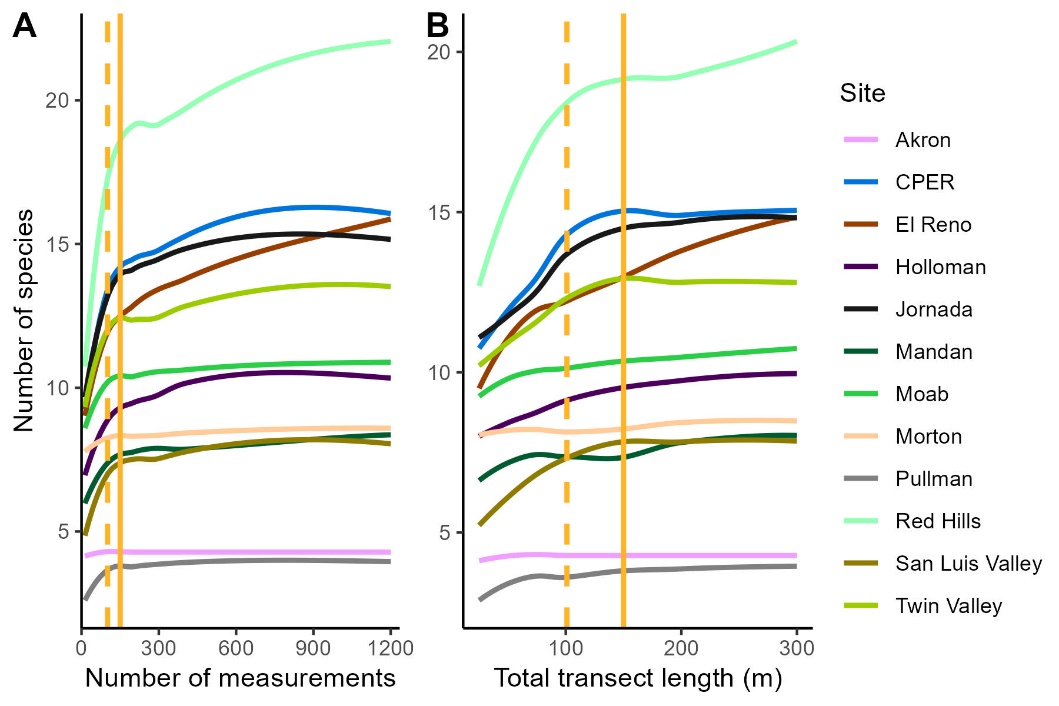


Figure S1.1. Species detection curves from the line-point intercept method for each National Wind Erosion Research Network (NWERN) site for increasing numbers of measurements (A) and total transect length (B).


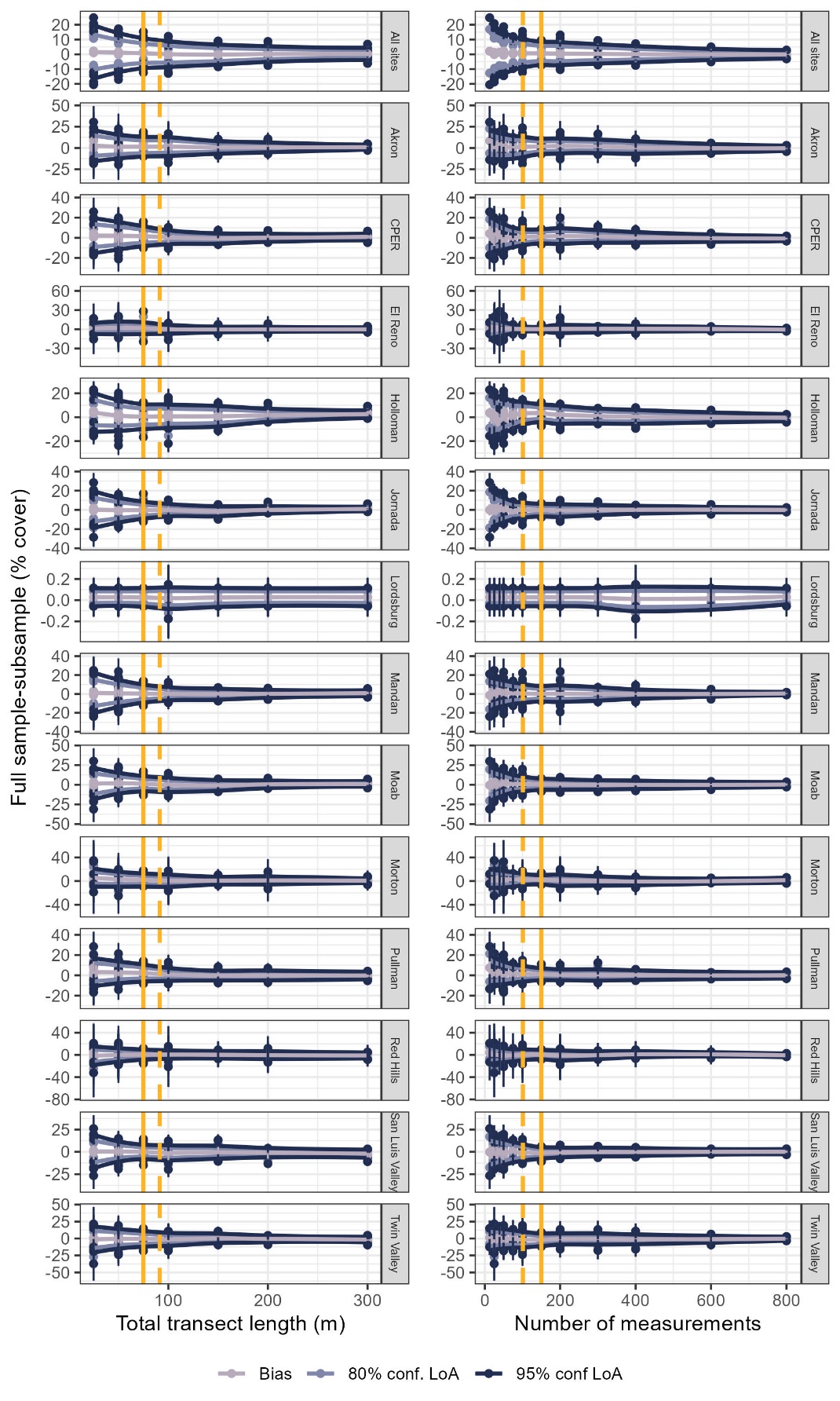


Figure S1.2. Limits of agreement intervals and bias for total foliar cover for different total transect lengths and numbers of measurements at 95% confidence level. The solid yellow line represents the sample design used by the Bureau of Land Management Assessment, Inventory, and Monitoring program. The dashed yellow line represents the sample design used by the Natural Resources Conservation Service
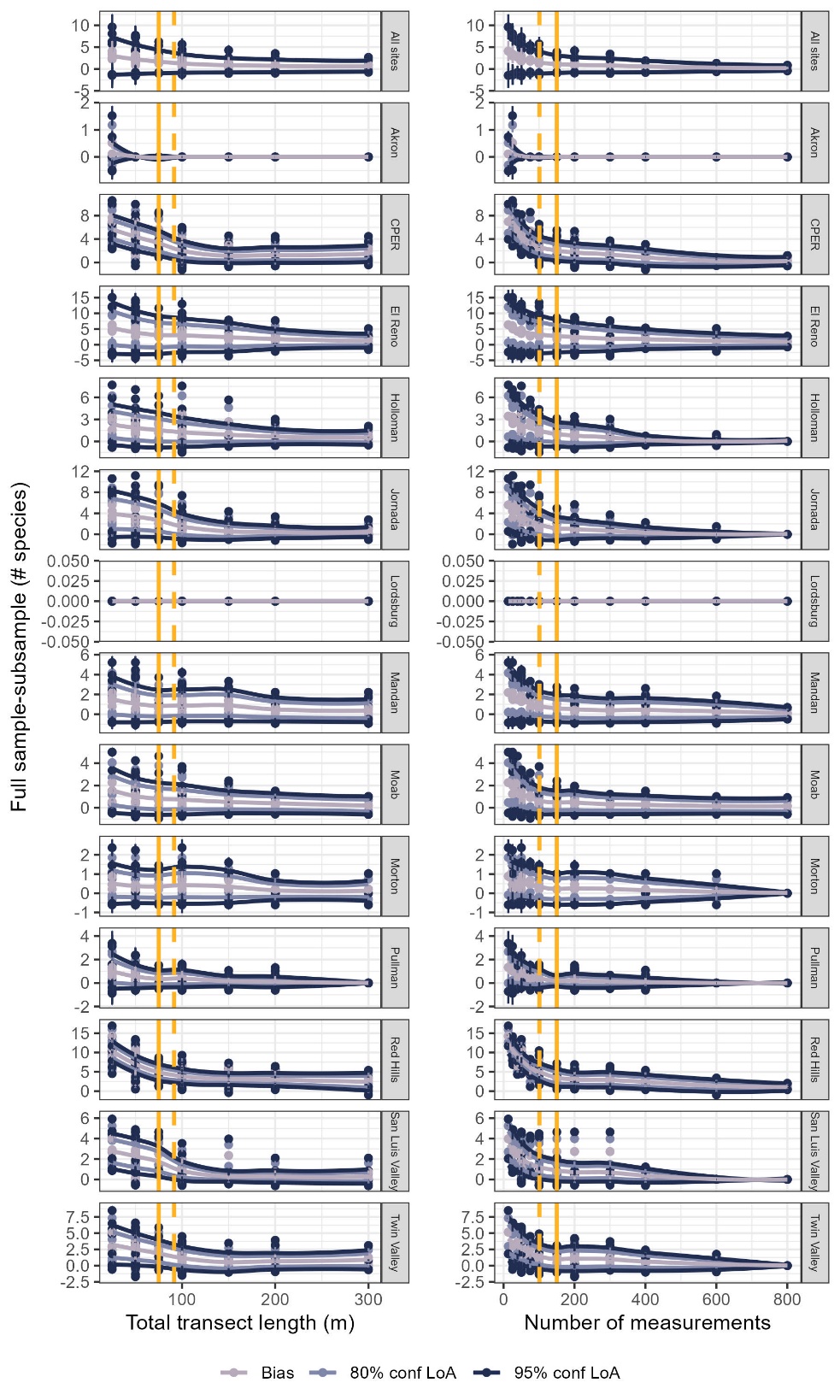
Natural Resources Inventory program.

Figure S1.3. Limits of agreement intervals and bias for species counts derived from the line-point intercept method at different total transect lengths and numbers of measurements at 95% confidence level. The solid yellow line represents the sample design of the Bureau of Land Management Assessment, Inventory, and Monitoring program. The dashed yellow line represents the sample design of the Natural Resources Conservation Service National Resources Inventory program.


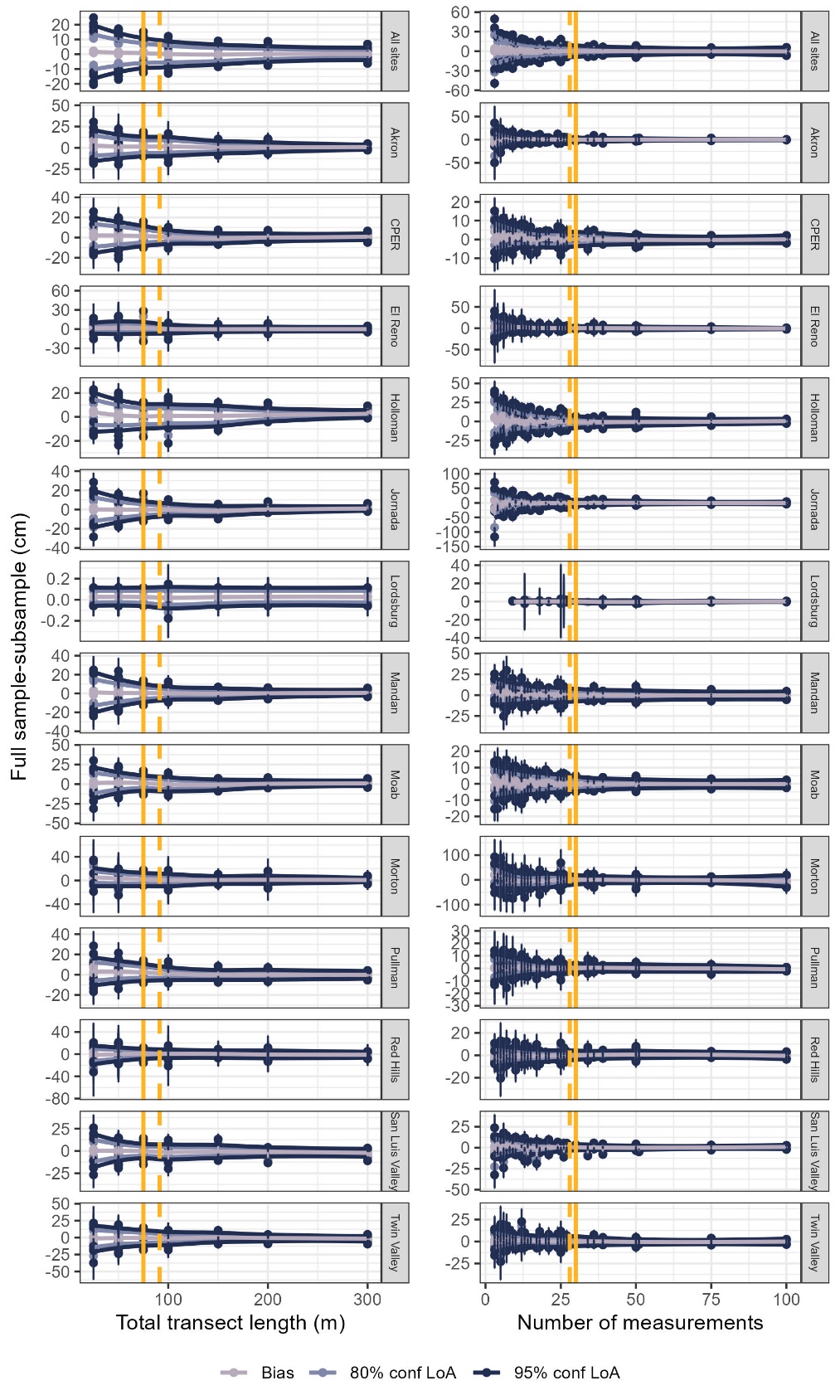


Figure S1.4. Limits of agreement intervals and bias for mean vegetation height derived from vegetation height method at different total transect lengths and numbers of measurements at 95% confidence level. The solid yellow line represents the sample design of the Bureau of Land Management Assessment, Inventory, and Monitoring program. The dashed yellow line represents the sample design of the Natural Resources Conservation Service National Resources Inventory program.


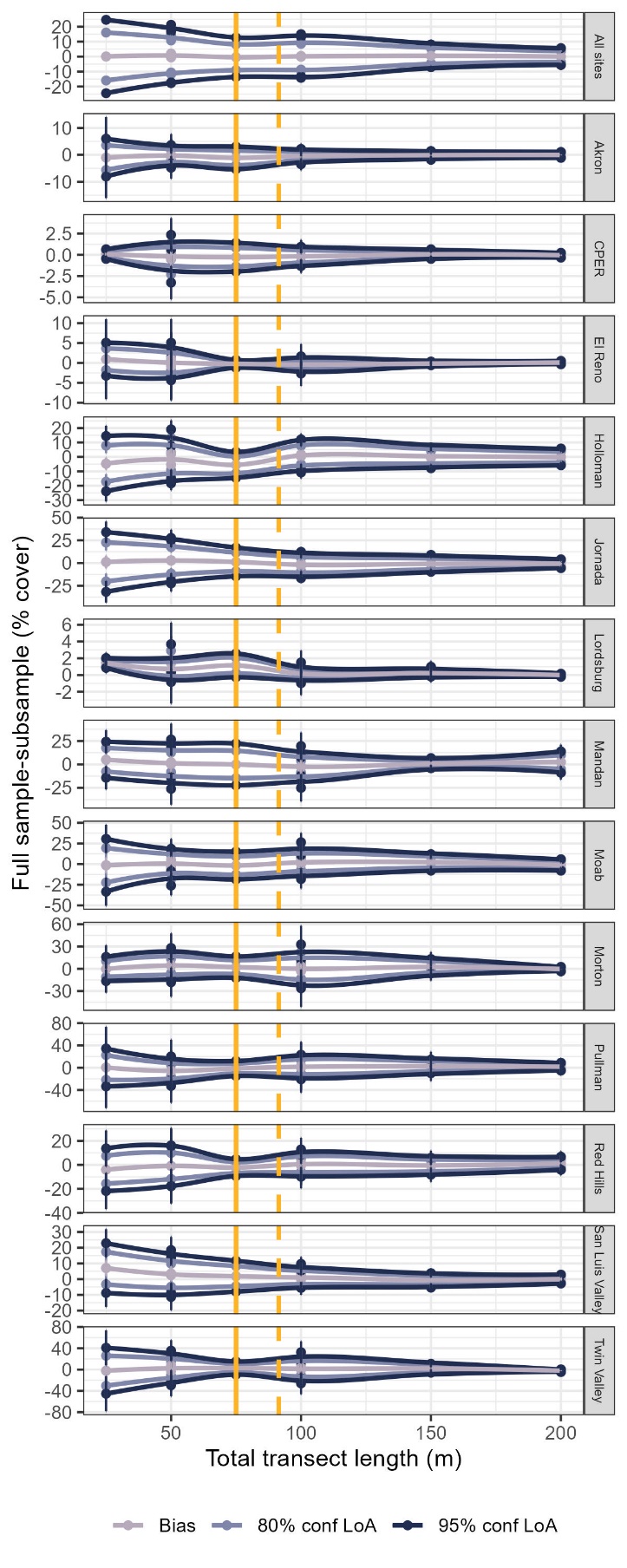


Figure S1.5. Limits of agreement intervals and bias for different total transect lengths at 95% confidence level for all-plant canopy gaps > 200 cm. The solid yellow line represents the sample design of the Bureau of Land Management Assessment, Inventory, and Monitoring program. The dashed yellow line represents the sample design of the Natural Resources Conservation Service National Resources Inventory program.
